# Gene-Gene-Environment Interactions of Serotonin Transporter, Monoamine Oxidase A and Childhood Maltreatment Predict Aggressive Behavior in Chinese Adolescents

**DOI:** 10.3389/fnbeh.2017.00017

**Published:** 2017-02-01

**Authors:** Yun Zhang, Qing-sen Ming, Jin-yao Yi, Xiang Wang, Qiao-lian Chai, Shu-qiao Yao

**Affiliations:** ^1^Medical Psychological Institute, The Second Xiangya Hospital, Central South UniversityChangsha, China; ^2^Medical College, North West University for NationalitiesLanzhou, China

**Keywords:** child abuse, MAOA-VNTR, 5-HTTLPR, aggression, gene × gene × environmental interaction, adolescent

## Abstract

Gene-environment interactions that moderate aggressive behavior have been identified independently in the serotonin transporter (5-HTT) gene and monoamine oxidase A gene (MAOA). The aim of the present study was to investigate epistasis interactions between MAOA-variable number tandem repeat (VNTR), 5-HTTlinked polymorphism (LPR) and child abuse and the effects of these on aggressive tendencies in a group of otherwise healthy adolescents. A group of 546 Chinese male adolescents completed the Child Trauma Questionnaire and Youth self-report of the Child Behavior Checklist. Buccal cells were collected for DNA analysis. The effects of childhood abuse, MAOA-VNTR, 5-HTTLPR genotypes and their interactive gene-gene-environmental effects on aggressive behavior were analyzed using a linear regression model. The effect of child maltreatment was significant, and a three-way interaction among MAOA-VNTR, 5-HTTLPR and sexual abuse (SA) relating to aggressive behaviors was identified. Chinese male adolescents with high expression of the MAOA-VNTR allele and 5-HTTLPR “SS” genotype exhibited the highest aggression tendencies with an increase in SA during childhood. The findings reported support aggression being a complex behavior involving the synergistic effects of gene-gene-environment interactions.

## Introduction

Teenage aggressive behavior is a major global public health problem. Longitudinal studies have indicated that aggressive behavior during youth is highly predictive of violence in adulthood and is also strongly associated with a greater risk of alcohol and drug abuse, violent crimes, suicide attempts and depression (Fergusson and Horwood, 1998; Bradshaw et al., 2010). The determinants of adolescent aggression are diverse and are known to include genetic as well as environmental factors. Numerous studies have shown that the interaction of certain genotypes and childhood maltreatment are linked to vulnerability towards aggressive or antisocial behavior (Caspi et al., 2002; Kim-Cohen et al., 2006; Byrd and Manuck, 2014; Haberstick et al., 2014).

Maltreatment of a child includes sexual abuse (SA), physical abuse (PA), emotional abuse (EA) and neglect (Miller et al., 2013). Such maltreatment is known to be a risk factor for adolescent aggressive behavior (Ford et al., 2012; Hoeve et al., 2015) and is associated with suicidal behavior (Carli et al., 2011), delinquency and violent criminal behavior in adults (Miller et al., 2013; Carli et al., 2014) as well as other psychological dysfunctional attributes in adulthood (Boyda and McFeeter, 2015). First, a lot of work has shown that emotional and mental health outcomes for adolescents who have experienced childhood abuse are worse than for those who did not. Furthermore, maltreated children are statistically more likely to engage in violent acts and enter the juvenile justice system than non-maltreated children (Lansford et al., 2007). However, a strong line of research has reported that several other forms of childhood adversity (such as stressful life events, family adversity, poor neighborhood, etc.) can also lead to negative effects on the mental health of adolescents (Hart and Marmorstein, 2009; Enoch et al., 2010). In terms of specific neurotransmitter effects, according to meta-analysis, the effects of monoamine oxidase A (MAOA)-environment interactions on antisocial behavior/aggressive behavior are more specific to child maltreatment than other early life adverse situations (Byrd and Manuck, 2014). Child maltreatment is a pervasive problem in Chinese society. According to a United Nations report, it was estimated that child physical abuse (CPA) experiences occur in about 10% of Chinese children (United Nations Children’s Fund, 2012). A meta-analysis of 47 studies on the prevalence of childhood PA in China found that the life time prevalence of any PA was significantly higher in the Chinese population than in others (Ji and Finkelhor, 2015). In addition, in another survey conducted on the Chinese mainland it was found that the prevalence of SA experiences before the age of 16 years among female and male college students were 24.8% and 17.6%, respectively (Chen et al., 2010).

According to the literature, childhood experience of maltreatment is the most critical environmental variable related to aggressive behavior in adolescents, especially within Chinese society. As a result, this was chosen to be the first selective environmental variable investigated in the present study. The strength of the correlation between specific forms of abuse and outcomes varies between different studies (Briere and Jordan, 2009; Wright et al., 2009). Indeed, many victims of childhood abuse are reported to experience, typically, more than one abusive event, placing them at a greater risk of re-victimization in adolescence and adulthood. Given the cumulative effects of various aspects of childhood trauma, there is a need to ascertain and delineate the specific associations between individual forms of childhood abuse and aggressive behavior.

Recent molecular genetic studies have revealed that many genes are associated with human aggression, such as: (1) serotonin (5-HT)-related genes; (2) dopamine-related genes; and (3) sex steroid-related genes. Of all these genes, serotonin-related genes have been studied the most. One gene that has been reported conclusively to influence aggression is the X-linked MAOA gene, which plays an important role in the degradation of central nervous system serotonin and norepinephrine. A deficiency in MAOA has been associated with aggressive behavior in adult males, and this is supported by studies in both animals and humans (Brunner et al., 1993; Cases et al., 1995). In healthy men, MAOA activity in the cortical regions correlate inversely with measures of aggression (Alia-Klein et al., 2008). MAOA-variable number tandem repeat (VNTR) has well-characterized VNTR functional polymorphism in the promoter region of the MAOA gene, which is located in the X-chromosome. There are five known variants, containing 2, 3, 3.5, 4 and 5 repeats of this sequence. A general consensus has been reached that the 2R and 3R alleles correspond to low MAOA activity, while the 3.5R, 4R and 5R alleles correspond to high MAOA activity as they exhibit more efficient transcription rates (Sabol et al., 1998).

The MAOA-L allele and environmental interactions on aggressive behavior/conduct disorder were firstly reported by Caspi et al. (2002). Since then the findings have been widely replicated in non-human primates (Newman et al., 2005; Pinto et al., 2010), human studies (Huizinga et al., 2006; Nilsson et al., 2006; Widom and Brzustowicz, 2006) as well as in laboratory paradigms of aggression (McDermott et al., 2009). In healthy male adolescents, low-activity MAOA-VNTR variants also predict lower spontaneous brain activity in the pons, which confers an individual’s risk for impulsivity and aggression (Lei et al., 2014). However, several other studies have reported that the MAOA-H allele is associated with an even greater propensity for antisocial behavior and impulsivity in males who experience early stress (Guo et al., 2008; Beach et al., 2010). Furthermore, the MAOA-H allele has been associated with impulsive personality traits in normal male subjects (Manuck et al., 2000). Apart from the differences in study designs, statistical analyses, outcome variables, environmental factors and their doses and valences, the failure to replicate gene-environment (G × E) interactions may be explained by the small effect a single gene has (Duncan, 2013), as well as possible gene–gene (G × G) interactions (Belsky and Pluess, 2009; Comasco et al., 2013; Goldman and Rosser, 2014).

According to a review (Popova, 2006), in addition to MAOA, serotonin transporter gene linked polymorphism (5-HTTLPR) in the serotonin transporter gene (SLC6A4) has been implicated as a strong candidate to be associated with violence. The 5-HTTLPR is a functional polymorphism in the promoter region of SLC6A4 which contains two common alleles: short (S) and long (L) variants. The short (S) 5-HTTLPR allele is less efficient transcriptionally than the long (L) allele (Lesch et al., 1996). A large number of studies have documented the involvement of this polymorphism in emotional dysregulation in humans (Ming et al., 2013, 2015). At the same time, genetic behavioral studies have led to suggestions that 5-HTTLPR is also related to a higher propensity for impulsivity and aggression in animals (Schwandt et al., 2010) and humans (Pavlov et al., 2012). It has been estimated that the presence of the SS-genotype can explain 5% of the inter-individual variance in human aggressive behavior (Preuss et al., 2005). Despite the inconsistent results (Liao et al., 2004; Sakai et al., 2006; Perroud et al., 2010), the S-allele of 5-HTTLPR has a significant association with increased aggression and impulsivity in various cohorts including children and adolescents (Beitchman et al., 2006), individuals with substance use disorders (Cao et al., 2013) and patients with personality disorders (Silva et al., 2010).

Findings so far, therefore, indicate that the genetic mechanism responsible for a predisposition to aggression and violence manifesting is an important goal in modern neurogenomics. A strong line of research has led to the suggestion that both MAOA and 5-HTTLPR have individual effects on human aggressive behavior. However, individual polymorphisms are likely to account for a relatively small amount of variance in a given phenotype (McGeary et al., 2012). Aggressive behavior is a complex trait, which is regulated by multiple genetic factors as well as environmental variables. Therefore, the examination of any single variant may be obscured when other relevant genetic variants are not included in the model simultaneously (Stuart et al., 2014). The preference of MAOA for the degradation of serotonin suggests that MAOA-VNTR and 5-HTTLPR may be mutually influential and may work collectively to affect a variety of behaviors and disorders. Furthermore, models of control systems have suggested that epistatic interactions between genes within components of the serotonin system are likely to have a role in aspects of 5-HT function. This, in turn, may be associated with aggression and impulsivity (Stoltenberg and Nag, 2010). Other experimental evidence derived from mice provides further support that there are interactions between these two genes (Murphy et al., 2003).

Given this evidence and the apparent multi-locus architecture of aggressive behavior, the present study was designed to investigate epistatic interactions among components of the serotonin system, especially MAOA-VNTR and 5-HTTLPR, which are already known to interact biologically. An attempt has also been made to examine the gene-gene-environment interactive effects involving MAOA–5-HTTLPR–childhood maltreatment in a group of otherwise healthy Chinese adolescents.

## Materials and Methods

### Participants

A total of 1248 healthy adolescents were recruited from four local middle schools in Changsha, Hunan Province China. Due to the uncertainty regarding the extent of X-inactivation at the MAOA locus, female adolescents (*n* = 647) were excluded from the following analyses with only male adolescents (*n* = 601) included. The inclusion criteria were: an ability to give voluntary informed consent; an absence of concurrent neurological or psychiatric disorders; no history of head trauma; no history of alcohol or drug abuse; no history of psychiatric illness or substance abuse on the basis of a SCID I assessment for DSM-IV criteria (First et al., 1997), and being of Han nationality. Among them, some participants missed the behavior measurement (*n* = 32), some failed to get DNA or genotyping (*n* = 13) and some other minority ethnics (*n* = 10) were also excluded from the study. So, eventually 546 male adolescents of Han nationality entered the analysis to be described below. The study was approved by the Ethics Committee of the Second Xiangya Hospital at Central South University in China, and all participants gave their written, informed consent to participate and were informed about the purpose of the study.

### Procedures

Trained researchers administered the questionnaires in the classroom. Every participant completed the following questionnaires: (1) Youth Self-Report (YSR) version of Children Behavior Check List (CBCL); and (2) Childhood Trauma Questionnaire–Short Form (CTQ-SF). At the same time, an exfoliated buccal sample from each of the participants was provided for DNA analysis.

### Behavioral Measurements

#### Aggressive Behavior

The CBCL is a 118-item parent report form, which has been designed to describe a 6–18-year-old child’s behavioral, emotional and social problems over the past 6 months (Achenbach, 1991). All items were rated on a 3-point scale: 0 (not true), 1 (somewhat or sometimes true) and 2 (very true or often true). The CBCL consisted of eight clusters of items representing common problems or syndromes identified by the following items: anxiety/depression, withdrawal/depression, somatic complaints, social problems, problems with thoughts, attention problems, rule-breaking behavior and aggressive behavior (Achenbach, 1991). Similarly to the CBCL, the YSR was a self-report style of questionnaire, which was developed to assess problems in youths aged 11–18 years old with the aim of yielding the broadband continuous dimensions of internalizing and externalizing symptoms. The YSR was normalized using a representative Chinese adolescent sample (of 12–16 year olds) and showed acceptable reliability and validity for mainland Chinese adolescents (Leung et al., 2006). The aggressive behavior subscale of the behavioral problems domain from the YSR self-report was used to measure tendencies towards aggressive behavior.

#### Childhood Maltreatment

Maltreatment was assessed using items from the CTQ-SF (Bernstein and Fink, 1998). This is a 28-item self-report inventory, which was developed to measure five types of abuse or neglect in childhood or adolescence. The five types are: SA, PA, EA, physical neglect (PN) and emotional neglect (EN; five items each), and an additional three items intended to measure any tendency to minimize or deny the abuse experience (the MD subscale). The CTQ-SF has been used widely in psychometric analyses. The internal consistency, stability over time and criterion validity of the current brief version has been established previously (Bernstein et al., 2003). Item responses were structured by a five-point, Likert-type scale ranging from never true (score = 1) to very often true (score = 5). Our CTQ data demonstrated high internal reliability of the PA, SA and EA subscales (Cronbach’s *α* = 0.806, 0.781 and 0.677, respectively). The PN and EN subscales showed low reliability and so were not used in the analysis. Cronbach’s *α* for the total maltreatment score, computed by summing the PA, EA and SA subscale scores, was 0.829.

### Genetic Analysis

#### DNA Collection and PCR Assays

Genomic DNA was extracted from exfoliated buccal cells using the TIA Namp Swab DNA Kit (TIANGEN Biotech, Beijing, China) according to standard procedures. Polymerase chain reactions (PCRs) were performed in a 25-μl reaction volume containing 1 μl DNA, 12.5 μl GoTaq Green Master Mix (Promega Company, Madison, WI, USA), 1 μl each of two primers (200 ng/l), and 9.5 μl ddH_2_O. The amplification protocol included cycling at 94°C for 3 min, followed by 35 cycles at 95°C for 30 s, 58°C for 30 s and 72°C for 45 s in a Gene Amp 2400 PCR system (Applied Biosystems, Carlsbad, CA, USA). PCR products were separated by electrophoresis on a 1.8% agarose gel and stained with Du Red (Biosharp, China), then visualized under ultraviolet (UV) trans-illumination. Sizes were determined by comparison with a 50-bp DNA sequencing ladder.

#### Genotyping

Because MAOA is located on the X chromosome, all male subjects were homozygotic for a single allele of the upstream VNTR. The two repeat and three repeat variants were commonly grouped as low-activity alleles and were contrasted with high-activity alleles of 3.5, 4 and 5 repeats, based on studies of MAOA promoter activity *in vitro* (Sabol et al., 1998). *5-HTTLPR* polymorphisms were determined using three genotypes: SS, SL and LL, as described previously (Lesch et al., 1996). According to the presence or absence of the 5-HTTLPR “L” allele, participants were assigned to the “SS” vs. “SL + LL” groups. Two independent allele classifications were carried out by two different individuals. In the case of a disagreement, a third investigator reviewed the classifications and samples were rerun as necessary.

### Statistical Analysis

Characteristics of study groups were compared using independent-sample *t*-tests. As the aggression scores measured by YSR were skewed, data were transformed before further analysis. The main effects of the MAOA-VNTR or 5-HTTLPR genotype, maltreatment and the two-way interactions of MAOA ×maltreatment (or 5-HTTLP × maltreatment), and the three-way interactions between MAOA × 5-HTTLPR × maltreatment were analyzed by using a linear regression model. All the maltreatment variables values (total maltreatment, PA, EA and SA) were centered before entering data into the model. All of the models included a correction for the effects of age.

## Results

### Characteristics of the Study Population

A total of 546 healthy male students (mean age = 15.6 years; standard deviation (SD) = 1.82 years) were included in the study. The low and high MAOA activity groups comprised 56.8% and 43.2% of the study population, respectively. 5-HTTLPR “SS”, “SL” and “LL” (*N* = 316/187/43) genotypes comprised 57.8%, 34.2% and 8.0% of the sample, respectively. The 5-HTT polymorphism was found to be in Hardy–Weinberg equilibrium (*χ*^2^ = 4.103, *p* = 0.128). Table 1 shows the mean scores and SDs of abuse and aggressive behavior. The demographic compositions of the genotype groups were comparable, with no difference in age or other family social status (Table 1). The *t-tests* demonstrated no differences in the total maltreatment and subscale scores of the CTQ questionnaire, or in aggression scores between participants with low and high activity MAOA-VNTR genotypes. A significant difference existed in the SA scores between the two 5-HTTLPR groups (“SS” vs. “SL + LL”) (*p* = 0.014), where the L allele carriers’ scores were reportedly higher than those of the “SS” genotypes. There were no other scores with significant differences identified between childhood maltreatment experiences and aggressive scores, as measured by the YSR (Table 1).

**Table 1:** **Characteristics of 546 healthy Chinese adolescents, according to genotypic classification**.

Measure (mean ± SD)	MAOA-VNTR			5-HTTLPR	
	Low activity *n* = 310 (56.8%)	High activity *n* = 236 (43.2%)	*p*	SS *n* = 316 (57.8%)	SL + LL *n* = 230 (42.2%)	*p*
Age (years)	15.6 ± 1.9	15.5 ± 1.7	0.853	15.6 ± 1.7	15.6 ± 2.0	0.976
Total abuse	20.4 ± 6.02	21.4 ± 7.3	0.090	20.5 ± 6.2	21.4 ± 7.0	0.118
PA	6.5 ± 2.49	6.9 ± 3.2	0.173	6.7 ± 2.9	6.7 ± 2.8	0.949
SA	6.3 ± 2.21	6.5 ± 2.9	0.358	5.2 ± 2.3	6.7 ± 2.8	**0.014**
EA	7.6 ± 2.91	8.1 ± 3.3	0.078	7.7 ± 2.8	8.0 ± 3.4	0.255
Aggressive behavior	11.6 ± 10.2	11.2 ± 7.24	0.633	11.5 ± 10.4	11.3 ± 6.8	0.771

*MAOA-VNTR, monoamine oxidase A variable number of tandem repeats; 5-HTTLPR, serotonin transporter linked polymorphic region. PA, Physical abuse; EA, emotional abuse; SA, sexual abuse*.

### Effects of Two-Way Interactions of MAOA or 5-HTTLPR and Childhood Abuse on Aggression

Linear regression results showed that total maltreatment and the three abuse subtypes were strongly associated with aggressive behavior, whereas MAOA-VNTR and 5-HTTLPR were not. There was no gene-environment interaction found between MAOA or 5-HTTLPR with any subscale of childhood maltreatment experience on adolescents’ aggression scores, as measured by the YSR scale (Tables 2, 3).

**Table 2:** **Results of linear regression model of the genetic factors (MAOA) and the subscale of childhood maltreatment associated with adolescent aggression**.

Aggression scores	*β*	*t*	*p*	AdjR^2^
MAOA (low vs. high)	−0.045	−1.028	0.280	
Total maltreatment	0.366	8.845	**<0.0001**	13.2%
MAOA × total maltreament	−0.10	−0.753	0.452	
PA	0.287	6.752	**0.005**	8.1%
MAOA × PA	−0.063	−1.00	0.318	
EA	0.326	7.776	**<0.0001**	10.5%
MAOA × EA	−0.043	−0.723	0.470	
SA	0.139	−1.0	**0.011**	4.9%
MAOA × SA	−0.031	−0.470	0.639	

*PA, physical abuse; EA, emotional abuse; SA, sexual abuse*.

**Table 3:** **Results of the linear regression model of 5-HTTLPR and the subscale of childhood maltreatment associated with adolescent aggression**.

Aggression scores	*β*	*t*	*p*	AdjR^2^
5-HTT (SS vs. SL + LL)	−0.042	−1.010	0.313	
Total maltreatment	0.366	8.845	**<0.0001**	13.2%
5-HTT × total maltreatment	−0.058	−0.990	0.323	
PA	0.287	6.752	**<0.0001**	8.1%
5-HTT × PA	0.011	0.206	0.837	
EA	0.326	7.776	**<0.0001**	10.5%
5-HTT × EA	−0.046	−0.760	0.447	
SA	0.227	5.247	**<0.0001**	4.9%
5-HTT × SA	−0.077	−1.20	0.231	

*PA, physical abuse; EA, emotional abuse; SA, sexual abuse*.

### Gene-Gene-Environment Interactions between MAOA-VNTR, 5-HTTLPR and Child Abuse on Aggression

**Figure 1:**
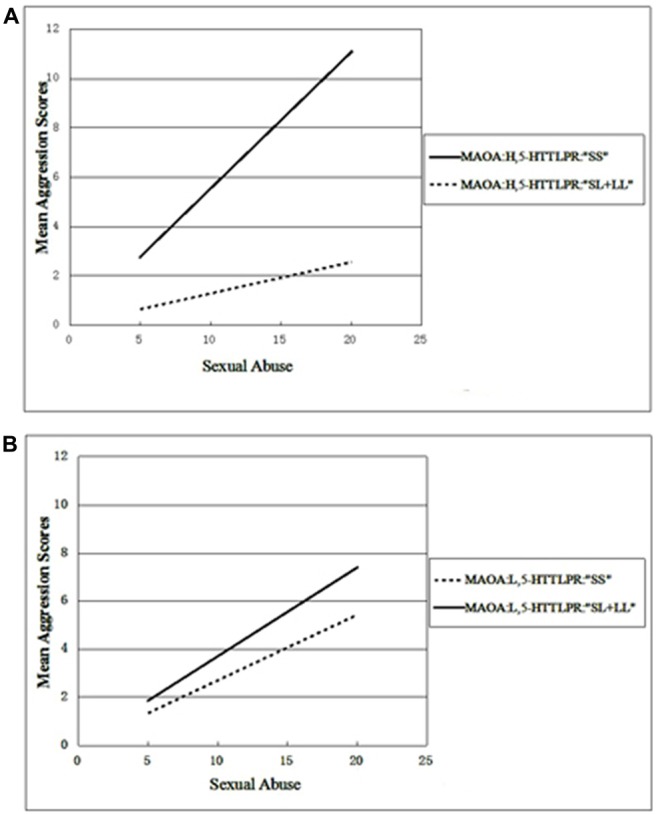
**Variation in the mean aggression scores with increases of sexual abuse (SA) and different gene combinations of monoamine oxidase A variable number of tandem repeats (*MAOA-VNTR*) and serotonin transporter linked polymorphic region (*5-HTTLPR)***. *MAOA*-H, high activity alleles; *MAOA*-L, low activity alleles; *5-HTTLPR* “SS”, homozygous short allele of 5-HTTLPR; 5-HTTLPR “SL + LL”; SS and SL genotypes of 5-HTTLPR. **(A)** The aggression tendency for boys with high MAOA activity with the increase of SA. **(B)** The aggression tendency for boys with low MAOA activity with the increase of SA.

Whether the three-way interactions between 5-HTTLPR, MAOA and the three subtypes of abuse could be associated with aggression was examined. To assess this, a linear regression model with two groups of the 5-HTTLPR genotypes (“SS” or “SL + LL”), MAOA (the L or H activity allele) and one of three subtypes of abuse (SA, EA or PA) were entered into the model, respectively. First, SA was included in the model, as a childhood maltreatment variable, and a significant three-way interaction between MAOA-5-HTTLPR-SA was found (*β* = −0.127, *t* = −2.458, *p* = 0.014). Next, PA and EA, as environment variables, were entered into the model. However, neither the interactive effect of MAOA × 5-HTT or the other three-way interactions were found to be significant (Table 4). To clarify the three-way interactions found between MAOA-5-HTTLPR-SA, all the adolescents were divided into low or high activity, according to their allele carrying status, and then the effects of 5-HTTLPR, SA and their interactions on aggression scores among these two groups were examined. When adolescents with low MAOA activity were included, a linear regression model showed no significant interaction between SA and the 5-HTTLPR allele. In contrast, for the high MAOA activity group, a significant interaction between SA and the 5-HTTLPR allele was revealed (*β* = −0.327, *t* = −3.483, *p* = 0.001). The *β* values were 0.554 and 0.129, for high activity MAOA carriers with 5-HTTLPR “SS” and “SL + LL”, respectively, and the *β* values were 0.271 and 0.317 for low activity MAOA carriers with *5-HTTLPR* “SS” and “SL + LL” (Figures 1A,B), respectively. These results suggest that the gene-environment interaction of *5-HTTLPR* × SA occurs primarily in the context of high MAOA activity. Male adolescents with a high MAOA activity allele and the “SS” genotype of *5-HTTLPR* exhibited higher aggression tendencies relative to their peers (who were carriers of other combined genotypes) with an increase in the experience of SA.

**Table 4:** **Results of the linear regression model of MAOA, 5-HTTLPR and childhood sexual abuse factor associated with adolescent aggression**.

Aggression scores	*β*	*t*	*p*	AdjR^2^
5-HTT (SS vs. SL + LL)	−0.045	−1.041	0.299	
MAOA	−0.028	−0.648	0.517	
SA	0.296	5.755	**<0.0001**	
5-HTT × MAOA	−0.050	−1.166	0.244	8.2%
5-HTT × sexual abuse	0.028	0.347	0.729	
MAOA × sexual abuse	0.096	1.198	0.231	
5-HTT × MAOA × SA	−0.127	−2.458	**0.014**	

*SA, sexual abuse*.

## Discussion

Aggressive behavior is one complex behavior, which is influenced not by one gene, but by many interacting genes. Therefore, a sensible rationale in searching for epistasis is to investigate gene networks, where several genes interact functionally. As the principal enzymes involved in serotonin metabolism, both the MAOA and 5-HTT genes are potentially of importance to understanding the occurrence of human aggressive behavior.

There is substantial empirical and theoretical evidence to imply variation in the function of the serotonin neurotransmitter system in the etiology of individual differences in behavioral control (Carver and Miller, 2006). Until now, evidence with respect to the role of gene–gene interactions in the serotonin system and (or) the dopamine system in the prediction of aggressive behaviors have been less well established (Nobile et al., 2007; Schmidt et al., 2007; Hohmann et al., 2009; Nilsson et al., 2015). There is even contradictory evidence of epistatic mechanisms being modified by environmental factors (Belsky and Pluess, 2009). Among the few studies on the epistasis or epigenetic effects among MAOA and 5-HTTLPR, some found a significant three-way interaction between MAOA, 5-HTTLPR, and family maltreatment on adolescents’ criminality/delinquency (Reif et al., 2007; Nilsson et al., 2015). In our study, we failed to find epistatic effects between MAOA-VNTR and 5-HTTLPR. However, a three-way interactive effect between MAOA-5-HTTLPR-SA was uncovered, which suggests that the epistatic effect might predispose individuals to aggressive behavior, but certain environmental triggers are also needed. The results also verify the assumption that aggressive behavior is a complex trait, which is regulated by multiple genetic factors as well as by environment factors that have a negative impact.

There is increasing evidence that early childhood trauma and abuse is associated with structural and functional changes in the brain, especially within the hippocampus, (medial) prefrontal cortex (mPFC), DLPFC and the amygdala. According to a review, children who have experienced early trauma or abuse exhibit decreased hippocampal volume (Glaser, 2000). Using MRI, Teichera et al. (2012) also found childhood maltreatment or abuse to be linked to a reduced adult hippocampal volume, particularly on the left side of the brain. At the same time, the development of the DLPFC may depend on healthy hippocampal development. It has been demonstrated that decreased DLPFC functioning is correlated with increased aggression, emotional reactivity and poor impulse control in adults (Coccaro et al., 2011). During emotional and neutral memory encoding and recognition, adults reporting childhood emotional maltreatment showed mPFC hypoactivity (van Harmelen et al., 2014).

It has also been reported that different forms of maltreatment experienced during childhood tend to be reflected in different types of criminal or violent behavior. SA was associated with an increased risk of pathological impulsivity and psychiatric disorders, including antisocial personality disorder and substance dependence (Braquehais et al., 2010), aggressive behavior and conduct disorder (Jonas et al., 2011). In contrast, individuals who had experienced other forms of childhood EA were more prone to developing depressive and anxiety disorders (Iffland et al., 2012; Nanda et al., 2016). Therefore, the effects of SA on an individual’s mental health may be different to those of physical or EA, and the specific mechanism underlying how this type of child maltreatment affects an individual’s behavior via variation of brain structure or function requires exploration as follow-up research.

It should be noted here that there was a significant correlation between the 5-HTTLPR genotype and children’s experiences of SA, in which boys with the “SL + LL” genotypes of 5-HTTLPR were more likely to have been exposed to a sexually abusive environment. However, this result would not interfere with the three-way interaction effect we found, in which individuals with a high MAOA-VNTR activity, and an “SS” genotype of 5-HTTLPR tended to manifest aggression tendencies most compared with other combinations of these two genotypes. So, this G-G-E interactive effect is unlikely to be a function of an evocative gene-environment correlation (r-GE). However, owing to the relative low AdjR^2^ of MAOA-5-HTT-SA found in our study (8.2%), further studies are needed to confirm our findings with a larger number of subjects.

In the present study, boys with the high* MAOA*-VNTR activity allele and the 5-HTTLPR “SS” genotype exhibited the highest aggressive tendencies with increasing childhood SA. This result appeared contradictory to the reports of Nilsson et al. (2015). In Nilsson’s study, boys from the two most genotypically divergent groups, MAOA-“LL” (high activity allele) + 5-HTT-“LL”+ BDNF Val-Val vs. MAOA“S/LS” (low activity allele) + 5-HTT-”S/LS” + BDNF Val-Met/ Met-Met), showed the highest criminality scores when they were exposed to environmental adversity such as family conflict/SA (Nilsson et al., 2015). The author suggested that some genotypes did not confer a risk in terms of delinquency but they rather altered an individual’s susceptibility to positive and negative environmental factors (Nilsson et al., 2015).

There were several reasons for interpreting these inconsistencies in this way. First, the behaviors investigated in these studies were phenotypically different from each other, even though they belong to the spectrum of externalizing behavior (Hohmann et al., 2009). We used aggressive behavior scores from YSR questionnaires as the outcome, while in Nilsson’s studies delinquency scores were used. Another possible explanation could lie in polygenics. All the genes mentioned above, as well as other genes, may contribute to aggressive behavior, each with a small individual effect. As all the results presented evidence for epistasis in the serotonin and/or dopamine pathways from neurotransmitter genes to behavior via brain circuits, the divergence of results of studies involving only one or two single genes interacting with the environment on an outcome variable may be partially explained. Furthermore, there is some evidence to suggest that serotonin related genes exert their effects at different stages of the serotonin pathway, so their effects may be cumulative and/or interactive (Bou-Flores and Hilaire, 2000). In association studies of complex traits, the concept of aggregate genetic risk scores (AGRS) was used to represent the potential summative effects of the “risk” allele (Stoltenberg et al., 2012). Linear genetic effects may not be strictly additive, but certain combinations of genotypes were associated with higher levels of psychopathological traits than would be expected with an additive model. For instance, previous research showed that an individual carrying a short 5-HTTLPR genotype and male carriers with low MAOA-VNTR variant activity exposed to a specific negative environmental factor were at a greater risk to a negative outcome. However, this did not mean that carriers of both these genotypes were at highest risk (Goldman and Rosser, 2014). Third, in our study, a group of Chinese male non-clinical adolescents were selected as the sample. Among them, the distribution of MAOA-VNTR was consistent with other studies conducted on the Chinese mainland showing that low activity carriers were much greater than high activity of MAOA (Chien et al., 2010; Qian et al., 2010), which was quite different from other reports focusing on Caucasian people. Among these, carriers with low MAOA activity were much less than the high activity group. So the different constitution of MAOA-VNTR among ethnic groups in these studies may also be an underlying reason for the discrepancies between study results. A final, but important, reason for inconsistencies could be that the different environmental factors and other characteristics of the subjects (demographic stratifications such as age, gender, healthy or clinical samples, ethnicity, sample size, etc.) varied in different studies, which are likely to either make direct contributions to behavioral phenotypes, or interact with genetic factors (Liu, 2011). However, given the general lack of evidence of epistaisis with environmental factors in this area, further efforts are needed to replicate this finding using other independent larger samples.

The current study has several limitations, which are significant enough to be mentioned. First, we focused only on healthy male high school students of Chinese Han nationality, so the results may not be generalizable to other populations. The situation in female subjects and the corresponding underlying mechanisms need to be explored in subsequent studies. Second, only 5-HTTLPR and MAOA-VNTR, two polymorphic genes, were selected for study. No other polymorphism in the serotonin and (or) dopamine system, which may also contribute to aggressive behavior were included (Beaver et al., 2007; Eisenberg et al., 2007; Guo et al., 2007). Hence, in future, a more extensive coverage of 5-HT system variations, according to HapMap information, may reveal more variants with significant effects. In addition, other neuronal systems such as the dopamine system may also contribute to aggressive behavior. Thus, this system should also be examined separately or in conjunction with the genes of the 5-HT system to determine the underlying neurobiological mechanisms of aggressive behavior. Finally, only childhood experiences of maltreatment were selected as the environmental variable, and no other sources of psychosocial adversity (such as family conflict or the quality of child-parent relationships), which may have a negative effect on an adolescent’s mental health, were included. Further studies will clearly be needed to determine the exact neurobiological mechanisms underlying the effects relating to comprehensive multi-gene and multi-environmental variables. While these limitations may be addressed by future research, the results presented here suggest that two genes within the serotonin system, as well as certain environmental factors may be implicated as contributing to aggressive adolescent behavior.

## Author Contributions

SY, JY and XW designed the study and wrote the protocol. YZ, QM and QC took part in data collection and experiment analysis. YZ, QM managed statistical analysis. YZ completed the literature searches and the first draft of the manuscript. All authors read and approved the final manuscript.

## Funding

This study is supported by grants from the National Key Technologies R&D Program in the 11th 5-year plan of China (Grant No. 2009BAI77B02) and the Natural Science Foundation of China (Grant No. 81471384).

## Conflict of Interest Statement

The authors declare that the research was conducted in the absence of any commercial or financial relationships that could be construed as a potential conflict of interest.
